# A Hospital as a Cinema-Theatre

**Published:** 1912-02-17

**Authors:** 


*To the Editor of* THE HOSPITAL.

### A HOSPITAL AS A CINEMA-THEATRE.

*To the Editor of* THE HOSPITAL.

SIR,—The article in your issue of February 10 is interesting but rather misleading so far as London is concerned. According to the London Building Act (Section 5, Subsection 27) a hospital is defined as a public building, and the term "dwelling-house" has no relation to it whatever, unless as probably in the case of a dwelling-house being adapted, say, for staff accommodation. Modern hospitals being divided into building units, it might be argued that the great hall or its equivalent is not part of the hospital proper under the Act, as it is under a separate roof. This contention would stand, but the great hall would still come under the classification in the same clause "used or adapted to be used for any other public purpose," as it is used for entertainments at Christmas, and the construction, including exit-ways, would be carried out according to the laws on the subject set forth in the several Buildings Acts. It is provided in the Cinematograph Act, 1909, Section 7, Clause 2, that where premises are used occasionally and exceptionally only for the purpose of such an exhibition, no licence is necessary for the premises, but notice should be given to the chief officer of police in writing seven clear days before the performance. The question is one that can only be decided on the merits of each particular case depending on very many points. The purpose of the Act is to provide safety for the audience in cases of panic, and so long as this is provided in a reasonable manner the law is satisfied. Regulations within the County of London are becoming more and more stringent in regard to public cinematograph theatres, and a very high standard is being evolved, and probably it is the reflex of this which is being felt in public institutions such as hospitals.—Yours obediently,

February 10. DISTRICT SURVEYOR.

